# Recent Advances of Fluid Manipulation Technologies in Microfluidic Paper-Based Analytical Devices (μPADs) toward Multi-Step Assays

**DOI:** 10.3390/mi11030269

**Published:** 2020-03-04

**Authors:** Taehoon H. Kim, Young Ki Hahn, Minseok S. Kim

**Affiliations:** 1 CytoDx, Seongnam-si 13486, Korea; thkim@cytodx.co.kr; 2 Biomedical Convergence Science & Technology, Industrial Technology Advances, Kyungpook National University, 80 Daehakro, Bukgu, Daegu 41566, Korea; 3 Department of New Biology, Daegu Gyeongbuk Institute of Science & Technology (DGIST), 333 Techno jungang-daero, Daegu 42988, Korea

**Keywords:** microfluidic paper-based analytical devices (μPADs), point-of-care testing, multi-step assay, fluid manipulation

## Abstract

Microfluidic paper-based analytical devices (μPADs) have been suggested as alternatives for developing countries with suboptimal medical conditions because of their low diagnostic cost, high portability, and disposable characteristics. Recently, paper-based diagnostic devices enabling multi-step assays have been drawing attention, as they allow complicated tests, such as enzyme-linked immunosorbent assay (ELISA) and polymerase chain reaction (PCR), which were previously only conducted in the laboratory, to be performed on-site. In addition, user convenience and price of paper-based diagnostic devices are other competitive points over other point-of-care testing (POCT) devices, which are more critical in developing countries. Fluid manipulation technologies in paper play a key role in realizing multi-step assays via μPADs, and the expansion of biochemical applications will provide developing countries with more medical benefits. Therefore, we herein aimed to investigate recent fluid manipulation technologies utilized in paper-based devices and to introduce various approaches adopting several principles to control fluids on papers. Fluid manipulation technologies are classified into passive and active methods. While passive valves are structurally simple and easy to fabricate, they are difficult to control in terms of flow at a specific spatiotemporal condition. On the contrary, active valves are more complicated and mostly require external systems, but they provide much freedom of fluid manipulation and programmable operation. Both technologies have been revolutionized in the way to compensate for their limitations, and their advances will lead to improved performance of μPADs, increasing the level of healthcare around the world.

## 1. Introduction

Paper-based diagnostic devices have been widely used for the past decades because of their capability to enable cost-effective point-of-care testing (POCT). In addition, their simple operation and capillary action without the need for external equipment for fluid transport have accelerated the spread of such technology. Besides these characteristics, POCT has been highlighted as an appropriate technology for developing countries with suboptimal medical conditions, as it enables cheap and quick diagnosis. Many diagnostic products, such as the dipstick test and lateral flow assay (LFA), have already been commercialized, which are of single-test type and do not require the injection of additional reagents. These single-step testing formats have been applied to conventional diagnostic applications, such as glucose, lactate, and uric acid assays [1]. However, many cases are not amenable to biochemical assays with simple paper-based devices. One of the main reasons is that many bioanalytical tests require multi-step assays that usually accompany various reagents and multiple washing steps, as well as the need to conduct liquid exchanges at specific locations and periods according to a protocol. Since George Whitesides’s group first introduced technology to form hydrophilic channels by patterning the hydrophobic material on a cellulose paper in 2007 [2], there has been a great advancement in fabrication methods, fluid control, and sensing technology to enhance diagnostic performance. Based on the technical maturation, relatively complicated assays, such as an enzyme-linked immunosorbent assay (ELISA) [3] and nucleic acid sampling testing (NAAT) [4], have been made on a paper-based platform. These developments can bring medical benefits to developing countries where medical technology and expertise are lacking, which, in turn, can resolve the international imbalance of medical benefits. Therefore, the realization of multi-step assays with paper-based diagnostic devices is very important to enhance the quality of medical welfare around the world.

Paper-based diagnostic devices for multi-step assays can be separated into three techniques: (1) creating channels on paper [5,6,7], (2) sensing technology [8,9,10], and (3) fluid manipulation technology [7,11,12]. First, forming a hydrophilic channel is essential for accurately delivering reagents and samples to the sensing area in the paper-based diagnostic device, and various methods for fabricating hydrophilic channels on paper have been developed as follows: (1) photolithography [13], (2) inkjet printing [14,15], (3) wax patterning [16,17], (4) plasma treatment [18], (5) paper cutting and shaping [19,20], (6) screen printing [21,22], (7) laser treatment [23], and (8) flexographic printing [24,25]. Among them, wax patterning technology has been the most commonly used by easy fabrication procedures. Applications using this technology have been expanded to various areas, such as medical diagnostics [26], food [27], environmental applications [28], biodefense [29,30], and drug abuse testing [31].

Second, the sensing technology for detecting analytes in samples is directly related to the performance of the diagnostic devices [8,9,10]. Desirable requirements of the sensing modules include high sensitivity and accuracy without external equipment, where the two factors usually undergo a trade-off relationship, so it is important to optimize these performances in assay conditions. The sensing approaches that are currently being used include (1) colorimetric methods [32,33,34,35,36], (2) chemiluminescence [3,37], (3) electrochemistry [38], (4) fluorescence [39], and (5) electrochemiluminescence (ECL) [40]. Since there are apparent pros and cons depending on the sensing method used, it is important to choose a sensing scheme appropriate for diagnostic specifications and applications. In addition, for more complicate analyses, including multiplex and multi-step assays, the number of assay reagents and washing steps will be increased, which will require a smart sensing technology to make possible multiplex detection. These technical streams will naturally make the geometry of paper channels and fluid manipulation technology critical.

Fluid manipulation technologies enable reagents and samples to be delivered to the corresponding regions in proper assay time. Fluid manipulation technology can be largely divided into passive and active valves by the presence of external inputs [41], and the performance efficiency of paper-based diagnostic devices can be increased by adopting appropriate valves according to the applications. Passive fluid control methods are used to manipulate the flow rate within a paper channel. They do not need external inputs or additional equipment. Thus, while passive valves are structurally simple and easy to manufacture, they are limited to precise fluid control and sometimes consume larger reagents and samples because of more complicated and longer paper channels. On the other hand, active fluid control methods typically rely on external inputs, which sometimes include manual operation by humans. Active valves have the advantages of versatile flow manipulation, programmability, reproducibility, the fast response time of valve on/off, and multiple operations; thus, they have more potential to satisfy the requirements of multi-step assays. However, in most cases, additional mechanical or external systems are required, which can give rise to deterioration of the core values of paper-based diagnostic platforms, simplicity, and cost-effectiveness. In this paper, we analyzed the research trends for paper-based diagnostic devices being researched and developed in terms of the fluid manipulation of paper microfluidics. Furthermore, we discussed the benefits and limitations of these technologies, as well as future perspectives, by introducing the application areas in which multi-step assay technologies are applied.

## 2. Fluid Manipulation Technologies in Microfluidic Paper-based Analytical Devices (μPADs)

A fluid manipulation technology, which can control the flow of reagents and samples, is the most important element in paper-based diagnostic devices with multi-step assays. This is because it allows the required reagents to flow selectively as needed, and fluid control is essential for multi-step tests using various reagents, such as antibody solutions, washing buffers, and substrate solutions. In order to implement multi-step tests in a paper microfluidic device, it is very important to understand the fluid’s behavior on paper. In paper microfluidic devices, the flow based on the capillary tube is governed by the capillary pressure:

$$P_{c}=\frac{2\gamma cos\theta }{R}$$

where *P_c_* is the capillary pressure, *R* is the radius of the capillary tube, *γ* is the fluid surface tension, and *θ* is the solid–liquid contact angle. Based on combination with Hagen–Poiseuille’s law, based on the assumptions of the fluid—laminar, incompressible, and Newtonian—the fluid in this paper follows the Lucas–Washburn equation [42,43] for a one-dimensional model in cylindrical tubes, which can be derived from

$$L={\left(\frac{\gamma Rcos\theta t}{2\mu }\right)}^{1/2}$$

where *L* is the fluid column length, and *μ* is the fluid dynamic viscosity. The capillary is the driving force for fluid to move into the tube, and the velocity of the fluid is reduced as the fluid penetrates into the capillary. More detailed discussions can be found in other reports [11,12,44,45].

**Figure 1:**
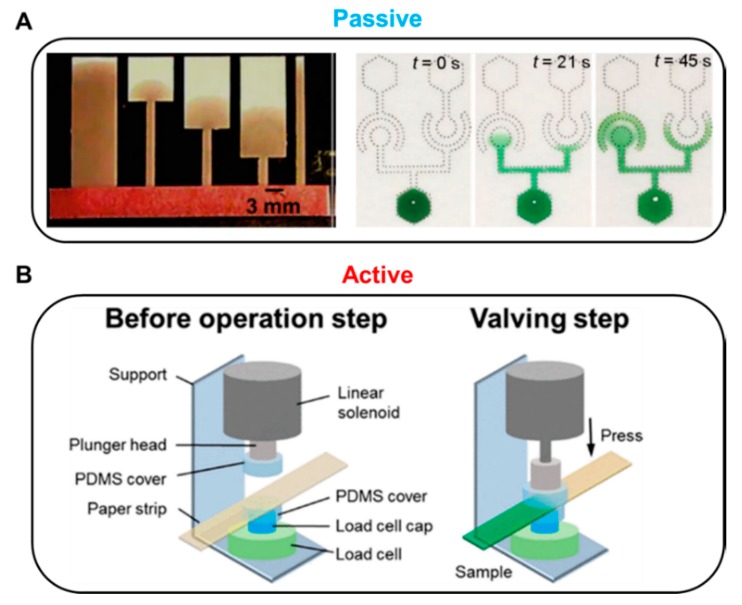
Representative methods for passive and active valves. (**A**) Passive methods. Geometry-based fluid control: Fluid delay time increases as the sudden expansion position on the strip moves away from the inlet. Figure reprinted with permission from [46]. Chemical-based fluid control: Principle of fluidic diodes using a surfactant. Figure reprinted with permission from [47]. (**B**) Active method: Solenoid-driven pressure valve pressurizes the paper, resulting in the disconnection of the fluid channels. Figure reprinted with permission from [48].

Fluid manipulation technology can be divided into passive and active methods according to the operation modes. In general, passive valves control fluids by changing the structure of the paper or by chemically treating the paper, while active valves use external inputs, including mechanical or electrical operation (Figure 1). A passive valve usually controls fluid flow by restructuring or chemical treatment of the device itself. In general, geometry and chemical-based fluid manipulation technologies are applied to passive valves. Passive valves are structurally simple and easy to fabricate but have a disadvantage of difficulty in terms of control fluid flow. A typical geometry-based fluid manipulation technology is to control the flow velocity by changing the position of the sudden expansion structure on the strip with the same width [46]. When the sudden expansion structure is close to the inlet, it shows a relatively short transport length compared to the far distance (Figure 1A, left). Such a valve is structurally simple, which is advantageous for mass production and cost-effectiveness; however, it presents difficulties in response speed control of the fluid because the fluid movement is controlled by the structure of a predetermined paper strip. Another passive valve technology is chemical-based fluid manipulation that works on the principle of fluid diodes using surfactants (Figure 1A, right). This method also simplifies the manufacturing process because it uses properties that can flow into the hydrophobic region as the surfactant dissolves [47].

In contrast, active valves primarily control the flow of fluid through geometry transformation and electrical or mechanical actuation. Mechanical-actuation-based fluid manipulation is one of the main active valve categories. For instance, solenoid-driven pressure valves delay or stop the flow of fluid depending on the pressure applied to the paper [48]. Applying voltage to the solenoid actuator causes the plunger head to pressurize the paper strip and stop the flow of fluid (Figure 1B). The active valve has the advantages of fast response, reproducibility, and compatibility with biological samples by no use of surfactants and chemicals. In addition, it does not require complicated and long paper channels frequently shown in passive valves. Since the pressure-driven valve system utilizes an Arduino microprocessor, it enables programmable fluidic control and repeatable valve operations. Despite its unique and versatile performance, a critical disadvantage is that the additional instruments, the solenoid valves, and the microprocessor make the device larger and increase the cost.

**Figure 2:**
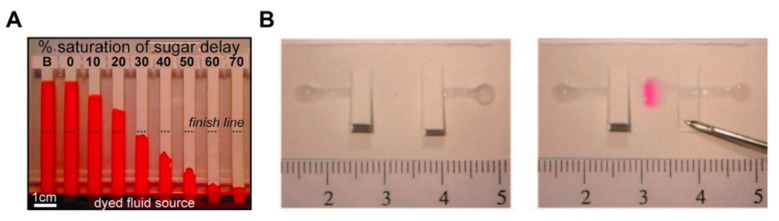
Classification according to the function of fluid manipulation technology. (**A**) Flow rate control using dissolvable sugar, which can control fluid velocities by adjusting the concentration of dissolvable sugar. Figure reprinted with permission from [49]. (**B**) Switching method using cut paper, which enables fluid flow by pressing the cut paper and connecting the passage of fluid. Figure reprinted with permission from [18].

A number of fluid control technologies based on the function of passive and active valves have been developed. Among other technologies, flow rate controlling and switching technologies have made a lot of progress. The controlling flow rate is a method of changing the velocity of a fluid on paper through structural, chemical, and mechanical ways. A representative method is to induce a programmable flow delay using dissolvable sugar (Figure 2A) [49]. This device uses various strips coated with 10%–70% dried sucrose to control the flow rate as intended. When the dried sucrose meets the fluid and dissolves, it increases the viscosity of the fluid and thus causes a fluid delay. Using this delay effect, multi-step testing is possible by adjusting the fluid velocity of each reagent on the paper. Unlike the flow rate controlling way, a switching method allows fluid to flow or not. A typical case is a way that allows fluid to flow by pressing a folded switch on a cut piece of paper to connect a separate fluid path (Figure 2B) [18]. This switching method enables multi-step assays through sequential reagent flow by turning each reagent flow on/off. The following sections examine in detail the functions and applications of passive and active fluid manipulation technologies.

### 2.1. Passive Fluid Manipulation

Paper-based analytical devices fundamentally exploit capillary action to transport the fluid. Although the control of fluid by relying solely on capillary action in μPADs is so passive that it limits the flexibility of fluid manipulation, it is still the most widely used method due to its simplicity, affordability, and expandability. The chemical treatment and geometry transformation of papers can be critical parameters in terms of fluid control in μPADs. A number of methods have been used to manipulate fluid delivery, including adjustment of the paper dimensions, control of the paper permeability, switching strategies, and change of the surface chemistry of the fluidic path on the paper.

#### 2.1.1. Chemical-Based Fluid Manipulation

Chemical-based fluid manipulation is a method of changing the wicking properties of paper by chemical treatment and can be divided into the fluid velocity control technique and the switching technique. Table 1 summarizes various chemical treatment-based fluid manipulation technologies for μPADs.

**Figure 3:**
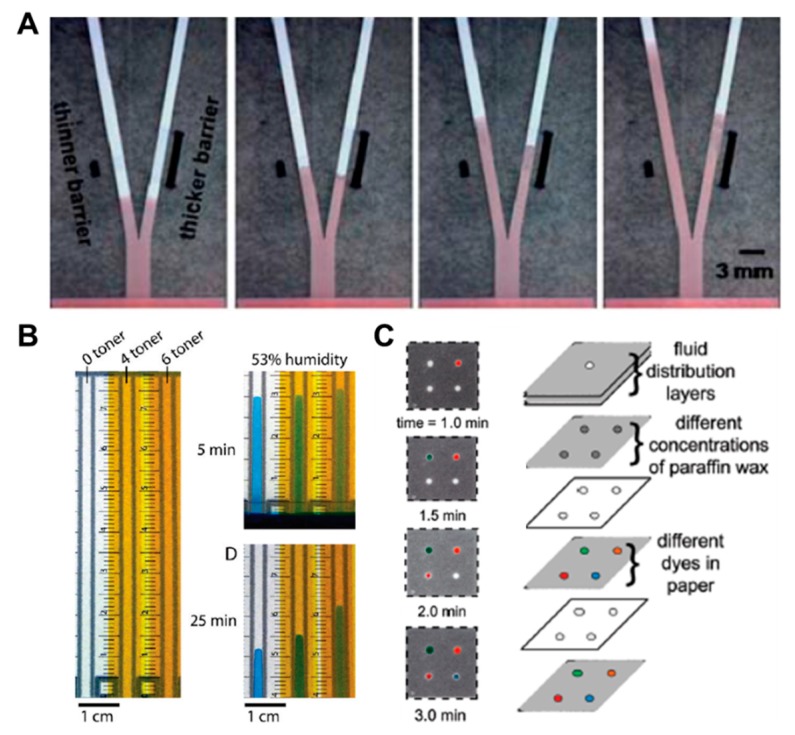
Fluid velocity control techniques by the coating of chemical materials. (**A**) Fluid manipulation through the different thickness of the dissolvable barrier. Figure reprinted with permission from [50]. (**B**) Fluid control in enclosed channels by toner printing. Figure reprinted with permission from [51]. (**C**) Flow rate adjustment technique according to paraffin wax concentration. Figure reprinted with permission from [52].

**Figure 4:**
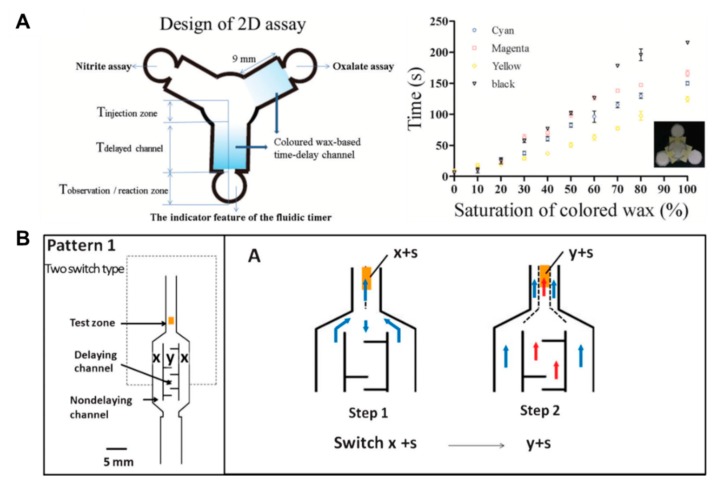
Fluid velocity control techniques by an inkjet printing method. (**A**) Control of wicking time according to the presence or absence of porous structures by wax types and contents. Figure reprinted with permission from [54]. (**B**) Fluid manipulation technique for multi-step assays by the inkjet printing method. Figure reprinted with permission from [55].

Above all, in the aspects of fluid control techniques, a great deal of research, including a method to delay the flow rate using dissoluble material (sucrose [49], trehalose [50]), has been conducted. A fluid delay channel is fabricated by injecting a diluted trehalose or sucrose solution into the strip and drying it. Then, when the injected sample reaches the dried dissolvable material, the dried material dissolves, and a fluid delay effect occurs by the dissolving time and the viscosity change. As shown in Figure 3A, trehalose is thickly dried on the right fork of the Y-shaped device and thinly dried on the left one. Subsequent injection of red dye into the inlet has resulted in a longer fluid delay time on the right fork. In addition, research on increasing the fluid velocity by patterning the toner several times on a paper strip through laser printing has been conducted [51]. On patterning the toner with hydrophobic properties above and below the channel, the flow rate increases by preventing the evaporation of a fluid, and it is also increased with the number of times of toner patterning. As Figure 3B shows, the flow rates have increased in the order of channels printed 6, 4, and 0 times under a 53% humidity environment after 25 min. Besides these technologies, methods of adding paraffin wax-coated layers on three-dimensional (3D) fluidic channels have been developed [52,53]. Coating hydrophobic paraffin on the channel can control the delay of fluid depending on the concentration. In Figure 3C, a fluid delay effect is achieved by stacking each micropatterned layer and interposing paraffin wax-patterned layers between them. In a similar way, Weng et al. studied the effect of fluid delay on the paper channel according to the color and saturation degree of colored wax [54]. Cyan- and yellow-colored waxes have a fine, porous-type structure when patterned on paper and have higher permeability than their black and magenta counterparts. Based on these characteristics, this group confirmed that adjusting the concentrations of colored wax controlled the flow rate delay effects (Figure 4A). As another approach using the patterning technique, a method of controlling the flow of fluid by forming a barrier pattern through inkjet printing on nitrocellulose paper has been studied [55]. Through various patterns, these devices can control the activation time by dissolving the dried reagents. In the case of Figure 4B, the sample flows to both the delaying and non-delaying channels, and the enzyme-linked detection antibody located in the non-delaying channel reacts with the analyte of the sample. Afterward, the enzyme-detection antibody complexes bind specifically to the capture antibody in the control and detection zones. On the contrary, the substrate on the delaying channel is rehydrated and switched from the sample fluid containing the previous complexes to the sample fluid, including the substrate (y+s), which allows the substrate to react with the enzyme-detection antibody complexes. This principle of operation enables sequential multi-step ELISA in a single step.

**Figure 5:**
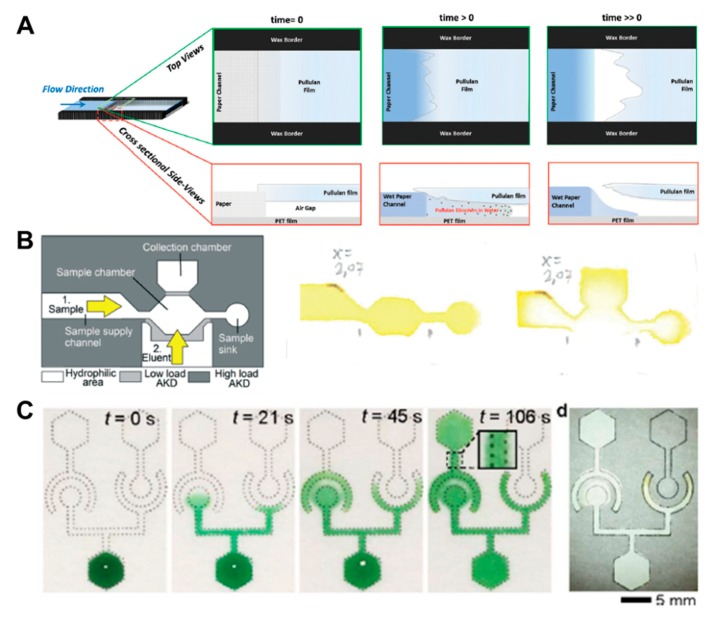
Fluid switching using chemical-based fluid manipulation technology. (**A**) Fluid switching using pullulan film. As the water fills the air gap between the pullulan and the PET (polyester) films, pullulan dissolves, and the capillary gap is destroyed, thus stopping the flow. Figure reprinted with permission from [32]. (**B**) Fluid control method using the dissolution of the alkyl ketene dimer (AKD) barrier by ethanol injection. Figure reprinted with permission from [57]. (**C**) Fluid diode technique based on surfactant. Figure reprinted with permission from [47].

In addition to fluid velocity control technology, switching methods using the principle of linking disconnected parts or blocking channels are also widely used in passive fluid manipulation technology. Research on a soluble bridge using the dissolving property of pullulan film and dried sugar has been carried out [32,56]. The soluble bridge is located between channels and acts as a temporal channel. After application of the sample, the bridge begins to dissolve slowly and functions as an on/off switch, when the volume of injected fluid reaches a certain amount (Figure 5A). However, this method has difficulty in precisely controlling the time when the bridge dissolves and breaks off. As another switching technique, a method to control the flow of fluids by alkyl ketene dimer (AKD), which is a hydrophobic material, has been developed [57]. The channel is patterned with high-load AKD, and the valve part that prevents the flow of fluid is patterned by low-load AKD. While high-load AKD retains its hydrophobic properties even with ethanol injection, low-load AKD loses its hydrophobic properties, allowing fluid to flow. Using this principle, it is possible to control the stepwise flow by opening the valve with an eluent (55% ethanol) after the application of the sample (Figure 5B). However, it is not guaranteed how the ethanol as an eluent will affect the sample.

Studies on fluid manipulation technology using surfactants have been conducted [47,58]. A fluid diode technology based on surfactant uses hydrophilic channels with dried surfactant made by the patterning of hydrophobic materials. When the sample is injected, the surfactant in the hydrophilic region dissolves. Consequently, the surface tension of the fluid decreases, and thus, the fluid flows into the hydrophobic region. The fluid diode has a specific structure in which the flow is blocked when the sample is introduced from the cathode side, whereas the fluid flows when the sample comes from the anode side (Figure 5C). The fluid control method with surfactants and dissolving materials, such as sucrose, has the advantage of being simple in structure, but using materials, such as ethanol, as mentioned in previous studies, may affect samples or downstream reactions. However, if verified materials, such as tween, which is often utilized in immunoassay using various antibodies, or sucrose, which is widely applied to dry and preserve antibody conjugates used in multi-step reactions, such as ELISA, are used, accurate assays can be performed without any significant impact on downstream action [49,61]. In addition, optimizing the deposition concentration of the surfactant or sugar has little effect on valve opening, on the solubility of each reagent, or on delivery capability, thus showing sensitivity that is equivalent to the existing methods [58]. In addition, several attempts have been made to control the flow of fluid through target-responsive hydrogels [59,60]. When aptamer cross-linked hydrogels encounter a target sample, they undergo the gel–sol phase transformation and flow. However, in the case of a fluid without the target sample, this phenomenon does not occur; thus, the hydrogel interferes with the flow of fluid in gel form. Based on this principle, hydrogels can be used as a switch to control fluid flow.

#### 2.1.2. Geometry-Based Fluid Manipulation

Geometry-based fluid manipulation technology is a method of adjusting the flow of fluid by altering the structural shape of the paper itself or by adding additional structures. There are representative methods of cutting, structural change, and attaching additional materials in this manipulation technology. Table 2 summarizes the studies on geometry-based fluid manipulation techniques for μPADs.

**Figure 6:**
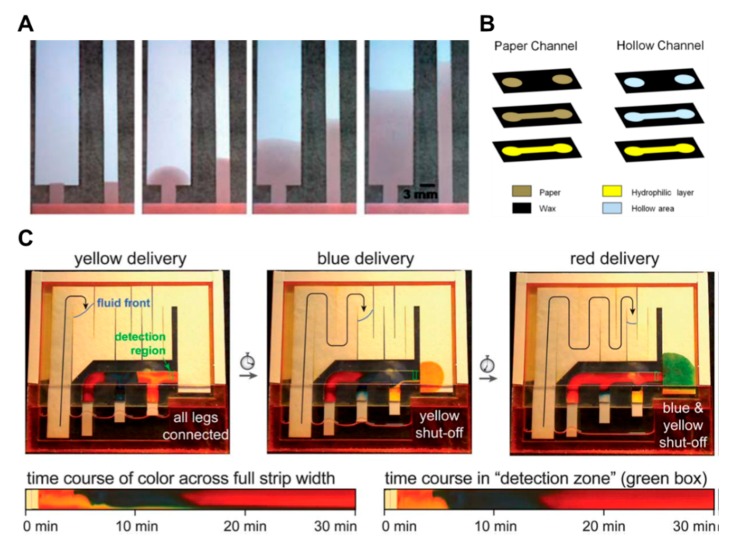
Geometry-based fluid manipulation technology using the cutting method. (**A**) The dependence of flow rate on the length and width of the paper strip. Figure reprinted with permission from [50]. (**B**) Hollow channel inducing fast pressure-driven flow according to the pressure of a single drop of liquid. Figure reprinted with permission from [34]. (**C**) Autonomous sequential fluid delivery in a 2DPN (two-dimensional paper network). The sequential fluids lead to continuous flow in the 2DPN. Figure reprinted with permission from [76].

Cutting the paper strip is a typical method in geometry-based fluid manipulation. A lot of studies have been conducted regarding how to control the flow rate by adjusting the width and length of the paper channel [44,46,50,62]. Figure 6A shows that the flow rate decreases as the width of the paper becomes wider. This is because the longer the width and length of the paper, the greater the resistance and the slower the fluid velocity. Other researches have attempted to increase the flow rate by adding a layer with hollow regions cut out in the form of a channel to a layer with an existing hydrophilic channel [34,35,38,64,65,66]. As shown in Figure 6B, the flow rate of the single drop of liquid injected into the hollow channel is about 7 times higher than that on a normal paper channel by forming a rapid, pressure-driven flow of about ~0.2 mbar [34].

The advantage of this approach is that the fast flow rate can reduce assay time and increase the size of the fluid network. In Figure 6C of the study, two-dimensional paper networks (2DPNs) have been applied to control the flow of fluid by varying the length of the leg in charge of each inlet [76]. When paper strips with different lengths are inserted into the fluid source wells, the fluid source of the wells is continuously reduced, and the shorter paper strips have briefer contact time with the fluid than the longer ones. This method has an advantage in that it is possible to make stepwise assays by making legs easily using cut paper and placing each leg into different reagent pads.

**Figure 7:**
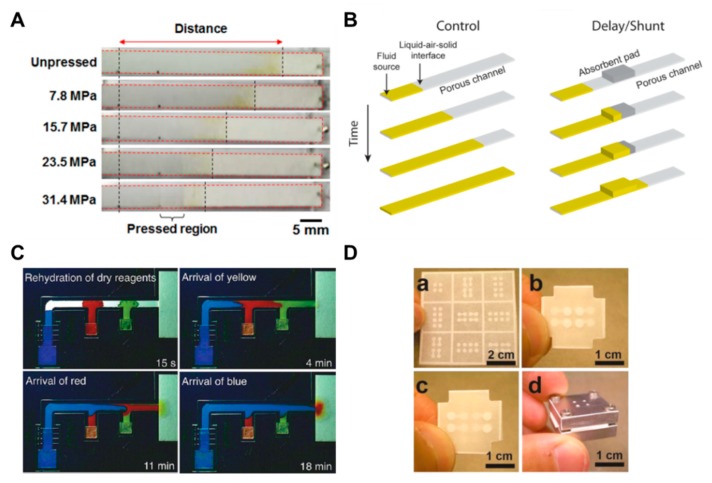
Geometry-based fluid manipulation technology using a method that changes structures and attaches additional materials. (**A**) A fluid velocity delay technique according to the pressure applied to the paper strip. Figure reprinted with permission from [67]. (**B**) A fluid delay technique using an absorbent pad (shunt). Figure reprinted with permission from [63]. (**C**) A 2PDN device that controls the flow of fluid through the change of acceptable fluid volume according to the size of the source pad. Figure reprinted with permission from [70]. (**D**) An origami device that changes the flow of fluid in a three-dimensional structure. Figure reprinted with permission from [73].

The methods using the structural change of paper or a combination of various kinds of papers and attaching additional materials have been frequently applied in μPADs. These methods have a principle that adjusts the flow rate by reducing the pore size and cross-sectional area on the paper by applying pressure to the paper [67,68,69]. If the pore size and cross-sectional area are reduced, the fluid resistance increases and, consequently, the flow rate decreases. As shown in Figure 7A, it is confirmed that the fluid transports differently depending on the pressure applied to the paper strips. The advantage of this approach is that a permanent fluid delay effect can be obtained by simply applying pressure to the paper. In another way, the use of a shunt, a method for controlling the flow rate by distributing the flow of fluid, has been studied [63]. This technique can distribute the flow of fluid by placing absorbent pads in the position where the fluid is to be delayed in the porous channel. In this study, it has been shown that the fluid delay time could be varied from 3 to 20 min by varying the thickness and length of the shunt (Figure 7B). Although this has a simple fabrication process, it has a limitation in terms of requiring a large quantity of sample due to the absorbent pads. In addition, a study has been conducted on how to control the flow rate by adding flexible films on the paper channel [33]. By sandwiching paper channels between two additional films, it prevents evaporation of the sample and thus accelerates the flow rate in comparison with channels without films. The advantage of this method is that the increased velocity of the fluid can reduce the diagnostic time.

Research has been conducted on technology that can control the maximum flow rate according to the source pad size [20,70,71]. Larger source pads can accommodate larger amounts of liquid and, therefore, have longer fluid transport distances and times (Figure 7C). Through this technology, stepwise assays can be applied to two-dimensional (2D) paper network chips, and reagent flow can be controlled by adjusting the size of the source pad. In contrast, methods of controlling the flow of fluid by stacking paper layers in a three-dimensional structure have been studied [72,73,74,75,77]. These methods use the principle of controlling the direction of fluid movement in the three dimensions by vertically connecting layers with hydrophobic regions (Figure 7D). This shows the characteristic of being able to assay target biomolecules by distributing the sample from one inlet to several outlets [72].

### 2.2. Active Fluid Manipulation

A lot of studies have also focused on active fluid manipulation, which is different from the passive fluid manipulation approach described in Section 2.1. While passive fluid manipulation is primarily utilized for uncomplicated assays, active fluid manipulation technology, which allows the fluid or sample to flow freely to the desired point at the desired time [78], can be applied to sequential multi-step assays. This active fluid manipulation approach makes simultaneous multiplex assays and multiple detection zones more affordable in μPADs. This technology can be classified into wettability-, geometry transformation-, and mechanical actuation-based methods.

#### 2.2.1. Wettability-Based Active Valves

Fluid control methods that use the chemical transformation of materials coated on the paper, for example, using the characteristics of corona discharge and electrowetting, have been developed and are summarized in Table 3.

**Figure 8:**
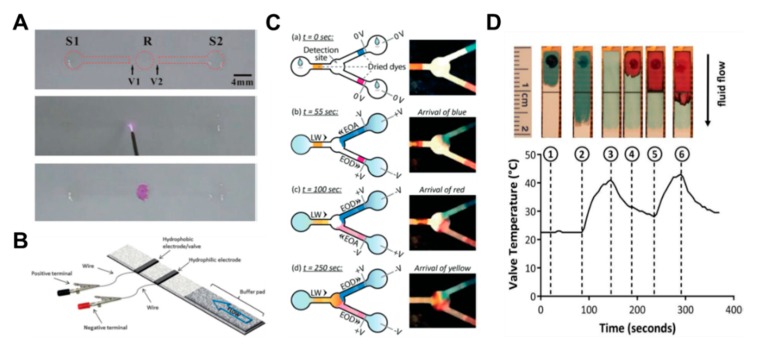
Wettability-based active fluid manipulation technology. (**A**) When corona discharge is applied to the valve part coated with octadecyltrichlorosilane (OTS), wettability changes, and the valve part regains its hydrophilic properties, and then the fluid can flow. Figure reprinted with permission from [79]. (**B**) When voltage is applied to the hydrophobic electrode printed by the inkjet technique, fluid can flow by restoring the hydrophilic properties due to the destruction of hydrophobic structures. Figure reprinted with permission from [80]. (**C**) Fluid velocity is controlled by using electro-osmosis when applying the electric field in paper-based devices. Figure reprinted with permission from [82]. (**D**) Wax-ink printing and localized heating via thin-film resistors to sequentially release liquids through a nitrocellulose membrane. Figure reprinted with permission from [85].

Some studies have been conducted on how to control fluids by converting the hydrophobic region into a hydrophilic property through corona discharge [79]. This method forms fluidic channels by coating octadecyltrichlorosilane (OTS) on all parts except the valving region and channel. Subsequent corona discharge treatments in the valving region allow this area to regain its original hydrophilic properties, enabling the flow of fluid (Figure 8A). A fast valve activation rate of about 1 s is an advantage, but the limitation is that it can be difficult to find the correct valve position and apply corona discharge. In addition to the corona discharge application, studies on valves using electrowetting, a phenomenon that can control the surface tension of a liquid by electricity, have been conducted [80,81]. The reason why surface tension of water changes on the application of electrical force is that the polarity of the water molecules creates a more attractive force for the conductive material flowing with electricity, which increases the surface tension. Koo et al. printed hydrophobic conductive electrodes (valves) on paper, together with hydrophilic conductive electrodes. The sample flows through a paper strip, passes the hydrophilic electrodes, and stops at the hydrophobic electrode. Then, applying a voltage across the electrode destroys the fluorinated monolayer of the hydrophobic electrode, causing the fluid to flow (Figure 8B).

Recently, a study on a fluid control method using electro-osmotic pumping has been conducted [82]. Applying a direct current voltage to electrodes on both sides of the wet paper causes the liquid to move toward one pole. This highlights the principle that the charge of the electric double layer at the solid–liquid interface is accompanied by the liquid when it is moved by the electric field (Figure 8C). This technology has advantages in that flow rate control, automatic control, and reversible (ON–OFF) actuation are possible. As another approach from the aforementioned method, research has been carried out on temperature control-operated valve systems [83,84,85], which is a method in which the flow of fluid is controlled according to the temperature change by using the characteristic that wax melts with temperature. In Figure 8D, the injected sample (blue liquid) flows to the outlet through the open valve over the wax patterned line (①,②). Heating the wax causes it to penetrate the channel and close the valve (③). At this time, the injected red liquid only flows to the closed valve and stops (④,⑤). When the heat is applied again, the wax melts, and the red liquid flows downwards (⑥) (Figure 8D). The characteristics of these valves are repeatable and can be automatically operated.

#### 2.2.2. Geometry Transformation-based Active Valves

A variety of techniques, including rotational valve, folding, push-button, sliding action, and paper switch, have been studied, and examples are shown in Table 4.

**Figure 9:**
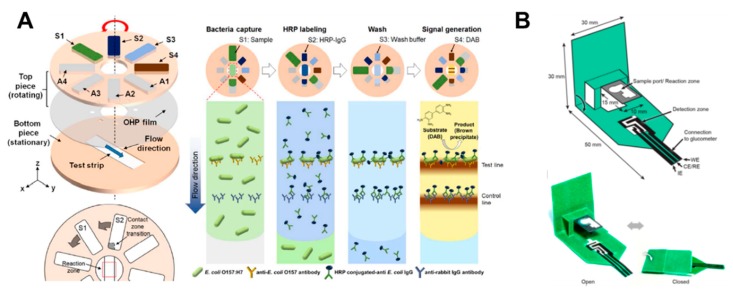
Geometry-based active fluid manipulation technologies using a rotational valve and folding methods. (**A**) Operation of the rotational valve. Each reagent flows toward the test strip step-by-step by sequentially returning the top piece on the test strip located at the bottom piece. Figure reprinted with permission from [101]. (**B**) A paper-based “pop-up” electrochemical device. By folding a device with a pop-up structure, the analysis is performed as the sample port contacts the detection zone. Figure reprinted with permission from [91].

Rotational valves on paper-based analytical devices have been implemented using rotatable disk-type packaging [101], hollow rivets [102], comb binding [103], and rotational paper-based microfluidic chips [39] as ways to bridge separated paper channels. As a representative case using rotational valves, a rotatable disk-type device has a bottom piece with a test strip and a top piece with a pad on which the reagent is dried. After dropping the sample on the test strip, it performs the assays by turning the bottom piece in the directions of S1, S2, S3, and S4, in that order, for stepwise reagent flow (Figure 9A). This rotational valve has the advantage of being relatively simple to operate but has a limitation in that each step must be manually operated. Researches on the technique of transferring one fluid flow to another channel by paper folding have been also conducted [29,37,86,87,88,89,90,91,92]. The “pop-up” device shown in Figure 9B has a structure wherein the sample zone touches the detection zone and performs electrochemical detection when the popped up paper is folded. This method has an advantage now that the working time of the valve is relatively simple to operate.

**Figure 10:**
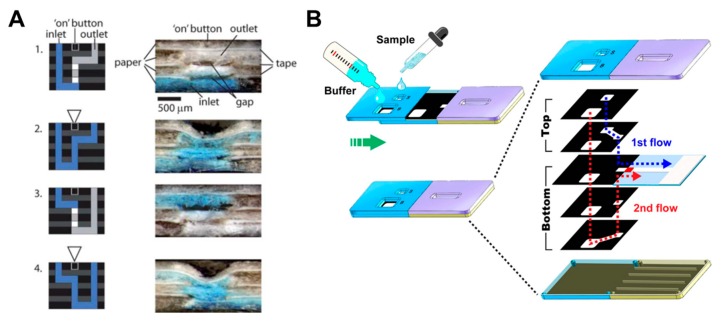
Geometry-based active valves using the push button and sliding action methods. (**A**) Working principle of the push button method. The three-dimensional stacking of perforated tape and wax-patterned paper allows fluid to flow by closing the gap when pressure is applied to the perforated area. Figure reprinted with permission from [93]. (**B**) Operation of the sliding action method. This device performs the assay by applying the buffer and sample to the sliding strip in advance and then sliding them. Figure reprinted with permission from [100].

In a manner similar to the paper folding method, Martinez et al. have developed a device with a push button and a 3D device structure that intersects paper layers with fluidic channels and tapes layers with holes [93]. When the hole is pressed by the button, the gap between the paper and the tape layers is reduced, and the fluid starts to flow (Figure 10A). This principle allows the switching of fluids to be a programmable operation but can be used only once. There have been many studies on sliding action valves [36,94,95,96,97,98,99,100], which is a method of flowing fluid in a way contacting the paper channels of two layers each other by sliding one of the different layers with paper channels. Han et al. flowed the sample and buffer, simultaneously, with the sliding of the top layer using 3D slip-PAD (paper-based analytical device) (Figure 10B), which allows starting the flow at the desired time with the sample and reagents prepared. Finally, the paper switch technique has already been discussed in the fluid manipulation chapter, explaining the switching function [18]. Manually pressing the cut and folded paper switch connects the disconnected passage of the fluid (Figure 2B).

#### 2.2.3. Mechanical Actuation-Based Active Valves

Fluid manipulation methods using mechanical movement include expandable materials, magnetic valves, pressure valves, and reconfigurable flow switches, and their examples are given in Table 5.

**Figure 11:**
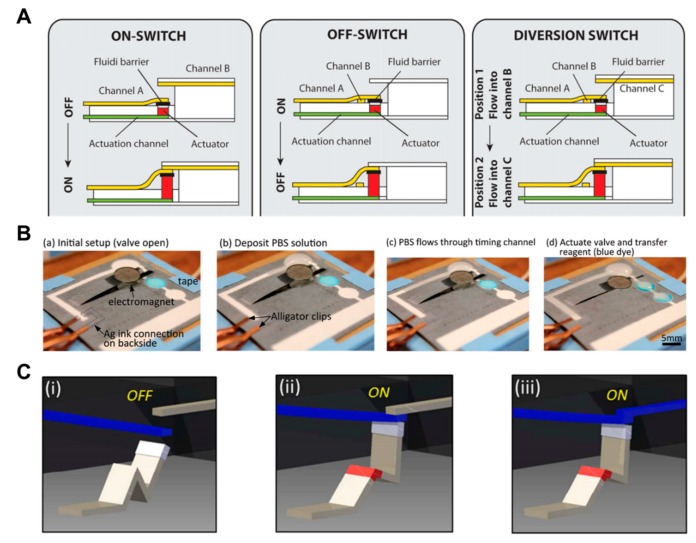
Mechanical actuation-based active valves, including expandable valves, magnetic valves, and reconfigurable flow switches. (**A**) Working principle of expandable valves. An expandable device expands according to the fluid flow and moves fluid channels to perform ON, OFF, and diversion functions. Figure reprinted with permission from [104]. (**B**) Operation of magnetic valves. This valve allows the fluid to flow by operating the cantilever valve with ferromagnetic nanoparticles, where an electronic device is located at the bottom. Figure reprinted with permission from [105]. (**C**) Operation of a reconfigurable flow switch. When the liquid is injected into the crest of the actuator made by folding the paper, it spreads out as it was before. It connects the input and output channels initially separated from each other. Figure reprinted with permission from [107].

Research has been conducted to control the flow of fluid using expandable material (sponge) [104]. When the sponge gets wet with the fluid flowing through the actuation channel, it expands so that the channel associated with the sponge transfers the flow of fluid to another channel or stops the flow. The advantage of this approach is that it can perform various functions (Off to On, On to Off, and diversion switch) (Figure 11A). An electromagnetic valve on paper-based analytical devices has also been studied [105,106]. The cantilever valve is made by applying ferromagnetic nanoparticles. In the case of a normally open valve, the cantilever valve remains folded, and when the electromagnet is operated, it is pulled toward the electromagnet to connect the separate channels. In this way, the flow of fluid can be automatically controlled (Figure 11B). This valve can be operated several times and is programmable [106].

A novel technique based on the pressure valve has been introduced as an example of an active valve in the fluid manipulation section [48]. Like a pressure valve using a solenoid actuator that can press the paper directly, it has the ability to decrease the flow velocity or blocking the flow according to the strength of the pressure (Figure 1B). The feature of this technology is that the valve can be operated repeatedly, and it is programmable and reversible. In addition to the above-mentioned techniques, research on the flow switch using the reconfigurable property of paper has been conducted [107]. The reconfigurable flow switch is a valve that uses the property of unfolding when liquid is injected into the folded part of the paper. When the liquid is injected into the actuator (folded paper) with the input and output channels structurally separated from each other, the tip pushes the input channel up and makes contact with the output channel (Figure 11C). This method is called single-pole single-throw (SPST) and can also be applied as single-pole double-throw (SPDT). The valve has the advantage of being able to operate only with paper and liquids, but it might have limitations in terms of mass production and complexity of the device. 

## 3. Applications

### 3.1. Nucleic Acid Amplification Testing (NAAT)

Molecular diagnostics is a technique used to analyze biological markers in the genome using gene amplification techniques, such as polymerase chain reaction (PCR). This method identifies a disease infection by extracting DNAs and RNAs containing the pathogen’s genetic information from the saliva and blood of the infected person and then amplifying them. The most popular method for molecular diagnostics is PCR, but it is limited because it requires expensive equipment with thermal cycling. However, with the development of isothermal amplification technology, DNA strands can be amplified without the thermal cycling process. The isothermal amplification method includes loop-mediated isothermal amplification (LAMP) [108], helicase-dependent amplification (HDA) [109], recombinase protein amplification (RPA) [110], and rolling circle amplification (RCA) [40]. Recently, several attempts have been made to transfer isothermal amplification of nucleic acid technology to the paper microfluidics platform for POCT [4,84,96,111,112,113]. These µPAD studies are summarized in Table 6 by comparing them with conventional methods.

**Figure 12:**
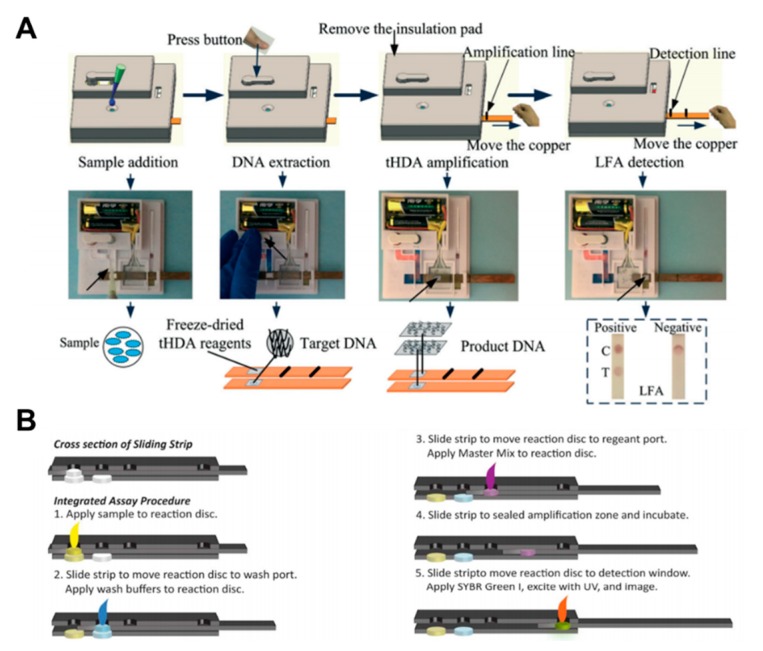
Nucleic acid amplification testing using paper-based analytical devices. (**A**) The whole operation process of the fully disposable and integrated paper-based sample-to-answer device. Figure reprinted with permission from [111]. (**B**) Operation processes of stepwise LAMP using a sliding strip device. Figure reprinted with permission from [96].

Isothermal amplification comprises three steps: sample preparation, nucleic acid amplification, and detection. Tang et al. implemented the whole operation process of HDA within an integrated paper-based platform [111]. After sample injection, the DNA extraction process starts by manually pressing the button. The strip is then moved over the thermal pad by a sliding action to carry out the amplification process. Finally, colorimetric detection is performed by moving the amplified sample to the test strip with another sliding action of the strip (Figure 12A). Connelly et al. implemented the LAMP amplification method on the sliding strip. After the sample injection into the reaction disc, the strip is placed in the wash port [96]. Then, the wash buffer is introduced, and the strip is moved again to the port for injecting the amplification master mix. As a final process, signals are obtained by exposing the strip to UV after injecting SYBR green I (Figure 12B). 

### 3.2. Enzyme-Linked Immunosorbent Assay (ELISA)

ELISA is a method of detecting and quantifying specific antigens or antibodies in biological samples, such as blood, urine, tissues, and cells. ELISA can be divided into direct ELISA, indirect ELISA, and sandwich ELISA according to the detection method. Sandwich ELISA is widely used because it shows higher specificity than the other methods. The processes of sandwich ELISA include stepwise reactions with several materials, such as the analyte, the enzyme-coupled detection antibody, and the substrate. The intermediate washing steps are also essential. Several studies have been conducted to implement ELISA in paper-based devices [36,55,58,120,121,122], as shown in Table 7.

**Figure 13:**
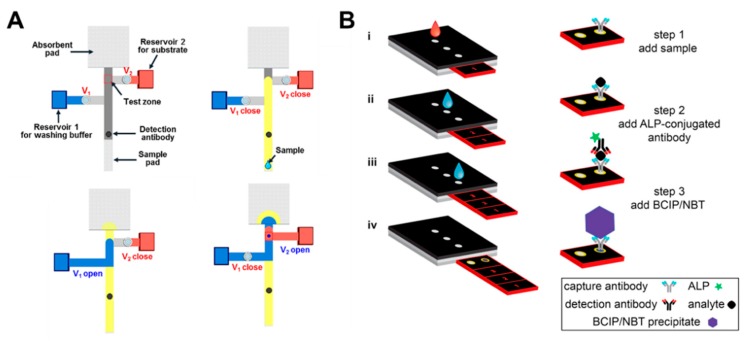
Applications of ELISA in a paper-based assay system. (**A**) Paper-based ELISA with pressure-driven valves (PDVs). Figure reprinted with permission from [48]. (**B**) C-reactive protein (CRP) detection device using a sliding strip 3D microfluidic paper-based analytical device (μPAD). Figure reprinted with permission from [36].

Our group recently developed a paper-based ELISA system using pressure valves [48]. The pressure valves of the system operate by a linear push–pull solenoid, which enables active control of the fluid flow on the paper strips. Based on this solenoid valve, ELISA with complicated multi-step procedures is successfully conducted. In the first step, when a sample is injected into the sample pad, the analyte of the sample reacts with the pre-dried detection antibody behind the sample pad (Figure 13A). The bound analyte–detection antibody then binds to the pre-dried capture antibody in the test zone. The next step is to wash off the unbound antibodies in the test zone by opening Valve 1 (shown as V_1_ in Figure 13A) to flow the washing buffer. Finally, colorimetric detection, depending on the concentration of analytes, is carried out by opening Valve 2 so that the color reaction of the enzyme in the test zone is induced. Verna et al. detected C-reactive protein (CRP) with ELISA using a paper-based 3D sliding strip [36]. This platform first injects the sample into the inlet and then moves the sliding strip to the inlet for alkaline phosphatase (ALP)-conjugated antibody injection. After the introduction of the ALP-conjugated antibody, the sliding strip moves to the inlet where 5-bromo-4-chloro-3-indolyl phosphate (BCIP)/nitro blue tetrazolium (NBT) is injected. BCIP/NBT is introduced, and the results are finally analyzed by removing the sliding strip from the device (Figure 13B).

### 3.3. Signal Enhancement Assay

A signal enhancement assay is a method to improve the detection signal and sensitivity. Because it requires several amplification steps (e.g., reagent injection and washing), it should be accompanied by fluid manipulation techniques for application in paper-based diagnostic devices. Thus far, several studies have been done on paper-based signal enhancement assays (Table 8).

**Figure 14:**
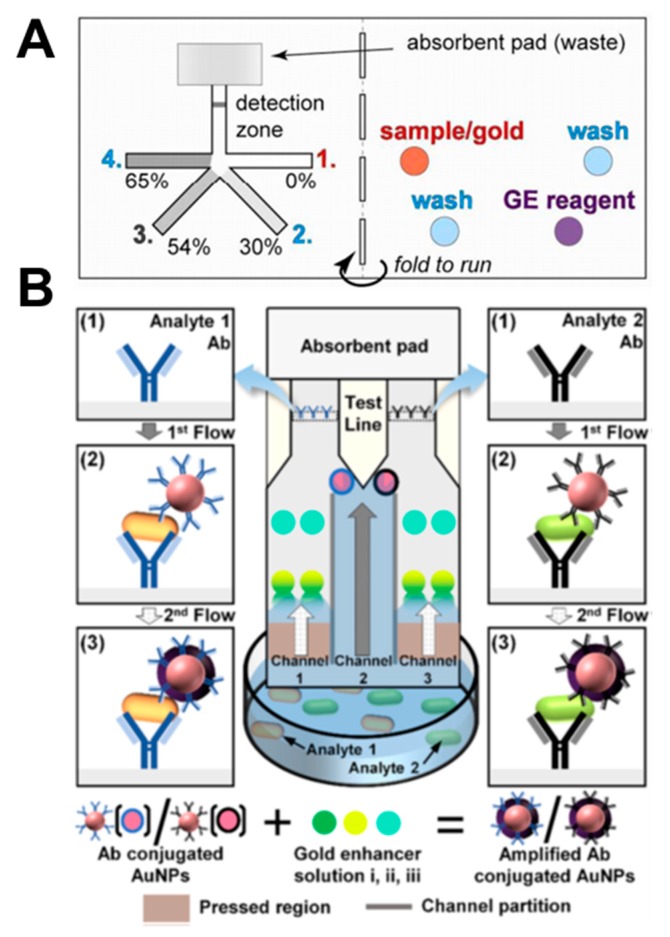
Applications of signal enhancement in paper-based analytical devices. (**A**) Stepwise signal enhancement method through paper strips using dissolvable material (sucrose). Figure reprinted with permission from [49]. (**B**) Stepwise operation of the pressed paper-based dipstick. Figure reprinted with permission from [68].

Lutz et al. performed stepwise signal enhancement through paper strips using dissolvable materials (sucrose) [49]. As shown in Figure 14A, four strands of strips are dried with 0%, 30%, 54%, and 65% sucrose, respectively. When the sample pad filled with sample, washing buffer, and gold enhancement reagent is folded and contacted with the strip, the velocity of the fluid is controlled according to the concentration of sucrose, and fluids reach the detection zone in the following order: sample, washing buffer, and gold enhancement reagent. Using this principle, they performed the PfHRP2 malaria assay. Park et al. enabled salmonella diagnosis through a step-by-step reaction using a pressed paper [68]. When the device is dipped into the sample, the analytes react with the antibody-conjugated gold nanoparticles (Figure 14B). Immuno-complexes are then captured by antibodies in the test line and give a colored reaction. The sample passing through the pressed region reacts with the pre-dried gold enhancer solutions i, ii, and iii and binds with the gold nanoparticles in the test line, resulting in an amplified signal. Through this method, amplified signals can be obtained from a mixture of *E. coli* O157:H7 and *Salmonella typhimurium*. 

Another approach to enhance sensitivity is to integrate an online sample stacking, enabling the use of a less sensitive detection modality [132]. Li et al. demonstrated a low-voltage paper isotachophoresis device for focusing on DNA samples [133]. By the 2-mm-long, 2-mm-wide circular paper channel formed by concertina folding a paper strip and aligning the circular paper zones on each layer, they could concentrate DNA with more than two orders of magnitude within 4 min. This electrophoretic approach makes possible the precise separation of mixed samples by adjusting the potentials applied at separation channels, not to mention the improvement of the limit of detection. While these studies usually utilize a relatively simple structure [134,135,136], they have the potential to realize in situ multiplex separation and detection among mixed samples with low cost, low-power requirements, and hand-held testing manners.

### 3.4. Colorimetric Enzymatic Assay

Colorimetric detection is the most widely used technology for paper-based analytical devices, which involves visual observation of the color change owing to the reaction of various reagents. This method enables either qualitative or quantitative analysis by taking advantage of the fact that it can be measured with the naked eye or with the help of simple visual readers. In particular, a colorimetric enzymatic assay using reactions depending on the concentration of analytes and enzyme, such as a glucose colorimetric assay, is a representative method in μPADs [34,60]. Glucose oxidase (GOx) and horseradish peroxidase (HRP) are commonly used to catalyze the reaction for glucose detection. The glucose catalytic reaction at the presence of glucose oxidase generates hydrogen peroxide (H_2_O_2_) and gluconic acid. Peroxidase then catalyzes the reaction of H_2_O_2_ with a color indicator, resulting in a color change. In this process, potassium iodide (KI) is the most commonly applied color indicator, resulting in a reaction showing brown coloration as KI is oxidized. In addition to the application based on glucose assay, colorimetric enzymatic assay in µPADs is widely used in various fields, such as pesticide assay [32,33], ion detection [79,103], and protein analysis [94]. These µPAD studies are summarized in Table 9 by comparing them with conventional methods.

**Figure 15:**
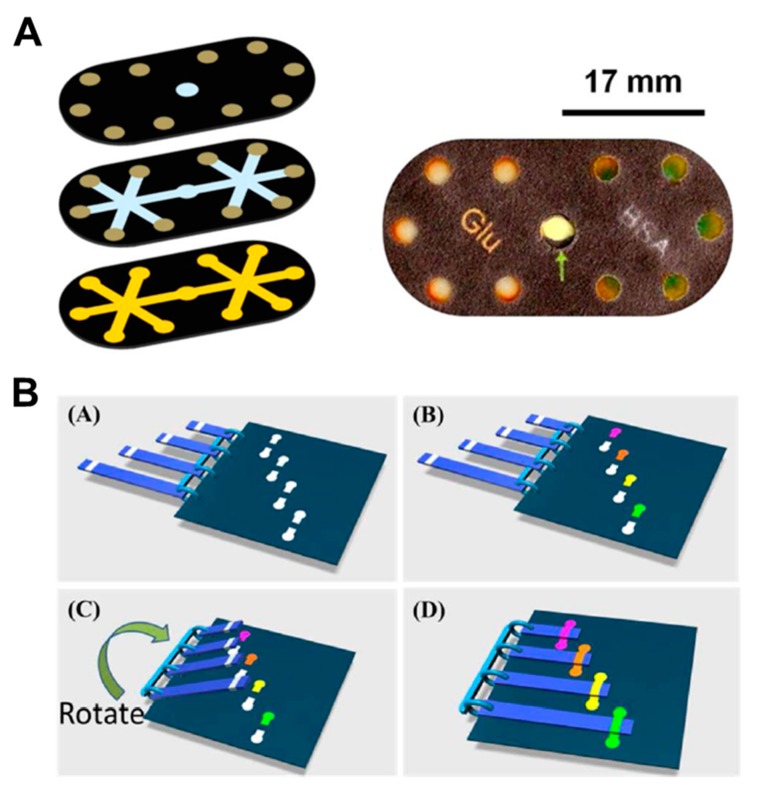
Paper-based colorimetric reaction method. (**A**) Components and resulting images of colorimetric glucose and protein assay using a hollow channel paper analytical device. Figure reprinted with permission from [34]. (**B**) Colorimetric tests using plastic comb binding spines (PCBS). Figure reprinted with permission from [103].

Renault et al. developed hollow channel paper devices for glucose and protein assays (Figure 15A). When the hydrophilic channel and the hollow channel are overlapped in a three-dimension, and the sample is injected into the inlet, the analyte reacts with the enzyme (KI, HRP) at the outlet, resulting in a color-producing reaction [34]. This combined structure increases the velocity of the fluid by about 7 times as much as that of regular paper channels. Han et al. tested the color reaction of Fe^2+^ and nitrite using plastic comb binding spine (PCBS)-based valves [103]. Although the inlet and detection zones have a gap distance between them, the analyte reacts with the reagents in the detection zone by connecting the strip between the inlet and the detection zone using a PCBS-based valve (Figure 15B).

## 4. Challenges and Perspectives

In recent years, many advances have been made in fluid handling in the field of paper microfluidics. Advances of fluid manipulation technology have led to improved performance and diverse applications of microfluidic paper-based analytical devices. In this paper, the fluid handling technologies were classified into passive and active methods according to the operation principle, which has advantages and disadvantages, respectively. Passive valves have the advantages of being able to reduce the cost and being more portable because the valve is configured in the device itself without any additional equipment. Based on the benefits, the approach is expected to evolve toward a way that can support faster response and higher integration with compact fashion. Active valves are advantageous for more accurate and programmable fluid manipulation because of the excellent responsiveness of the valves. However, since active valve methods are accompanied by additional equipment, cost-effective and straightforward modules will be desirable. In terms of practical utility and commercialization, the delicate calculation will be required between the cost increase by additional active valve modules and the expected values by reduction of reagents and performance enhancement. What is clear is that if the value of lateral flow assays—a simple, convenient, and cheap diagnosis—itself is fading away, it will face practical limitations no matter how sophisticated the active valve technology is. As such, it would be desirable to conduct technical development within the scope of the original value of paper-based platforms.

The potential requirements of fluid manipulation technologies for multi-step assays in paper-based analytical devices can be summarized as follows: (1) compact form, (2) instant response, (3) repeatable use, and (4) automated operation. The development of paper-based multi-step assay technologies through fluid handling techniques can bridge the current disparity in medical advancements globally. For instance, complicated assays for many viral and infectious diseases can be performed on-site in undeveloped countries if the assays currently being performed in the laboratory are implemented in paper-based technology. Therefore, it can accelerate the time to commercialization if it (1) meets the ASSURED (Affordable, Sensitive, Specific, User-friendly, Rapid and Robust, Equipment-free, and Deliverable) criteria of the World Health Organization (WHO), which is the standard of point-of-care testing and, at the same time, (2) solves the challenges with full automation and obtains high sensitivity of paper-based diagnostic technology capable of multi-step assays. In addition, providing medical benefits to developing countries can greatly contribute to the diagnosis of diseases caused by super bacteria or viruses and can help prevent disease spread.

## Figures and Tables

**Table 1:** Chemical-based fluid manipulation techniques for microfluidic paper-based analytical devices (μPADs).

Method	Function	Application	Ref.
Dissolvable material (Sucrose)	Fluid velocity control	Signal enhancement assay (^#^ PfHRP2 malaria assay)	[49]
Dissolvable material (Trehalose)	Fluid velocity control	-	[50]
Toner (laser printer)	Fluid velocity control	Enzymatic–colorimetric assay (glucose and alkaline phosphatase; ALP)	[51]
Parraffin wax	Fluid velocity control	-	[52]
Parraffin wax	Fluid velocity control	Enzymatic–colorimetric assay (glucose)	[53]
Colored wax	Fluid velocity control	Enzymatic–colorimetric assay (nitrite/oxalate assay)	[54]
Inkjet printing	Fluid velocity control	ELISA (human chorionic gonadotropin; hCG)	[55]
Dissolvable bridge (sugar bridge)	Switching (OFF)	-	[56]
Dissolvable bridge (pullulan)	Switching (OFF)	Enzymatic–colorimetric assay (Malathion)	[32]
Alkyl ketene dimer (AKD)	Switching (ON)	-	[57]
Surfactant	Switching (ON)	Enzymatic–colorimetric assay (ALP)	[47]
Surfactant	Switching (ON)	ELISA (Rabbit IgG)	[58]
Target responsive hydrogel	Switching (ON)	Enzymatic–colorimetric assay (Pb^2+^, Cocaine, and adenosine)	[59]
Target responsive hydrogel	Switching (ON)	Enzymatic–colorimetric assay (Cocaine, adenosine, uridine, and cytidine)	[60]

^#^ PfHRP2: *Plasmodium Falciparum* histidine-rich-protein-2.

**Table 2:** Geometry-based fluid manipulation techniques for μPADs.

Method	Function	Application	Ref.
Changing the area of the channel	Fluid velocity control	-	[44,46,50,62]
Shunt	Fluid velocity control	Signal enhancement assay (malaria protein PfHRP2)	[63]
Covered film	Fluid velocity control	Colorimetric–enzymatic assay (Malathion)	[33]
Hollow channel	Fluid velocity control	Colorimetric–enzymatic assay (Glucose and protein)	[34]
Hollow channel	Fluid velocity control	-	[64]
Hollow channel	Fluid velocity control	Colorimetric–enzymatic assay (Fe^3+^, Ni^2+^)	[35]
Hollow channel	Fluid velocity control	Electrochemical detection (FcMeOH)	[38]
Hollow channel (with triboelectric effect)	Fluid velocity control	-	[65]
Hollow channel	Fluid velocity control	Colorimetric–enzymatic assay (glucose, albumin, pH)	[66]
Pressurized paper	Fluid velocity control	Signal enhancement assay (progesterone receptor)	[67]
Pressurized paper	Fluid velocity control	Signal enhancement assay (*Escherichia coli* O157:H7 and *Salmonella typhimurium*)	[68]
Pressurized paper (with three-dimensional stacking)	Fluid velocity control	Signal enhancement assay (C-reactive protein)	[69]
Source pad of different sizes (^#^ 2DPNs)	Fluid velocity control	Signal enhancement assay (BSA-biotin)	[20]
Source pad of different sizes (2DPNs)	Fluid velocity control	Signal enhancement assay (malaria protein PfHRP2)	[70,71]
Three-dimensional device	Fluid velocity control	Enzymatic assay (glucose and protein)	[72]
Three-dimensional device	Fluid velocity control	Colorimetric–enzymatic assay (protein)	[73]
Three-dimensional device	Fluid velocity control	Immunoassay (malaria protein PfHRP2)	[74]
Three-dimensional device	Fluid velocity control	Colorimetric–enzymatic assay (glucose)	[75]
Vertical paper legs of different lengths (2DPNs)	Fluid velocity control	-	[76]
Three-dimensional device	Fluid velocity control	Colorimetric–enzymatic assay (glucose, albumin)	[77]

^#^ 2DPNs: Two-dimensional paper networks.

**Table 3:** Wettability-based active fluid manipulation techniques for μPADs.

Method	Function	Application	Ref.
Corona discharge treatment	Switching (ON)	Colorimetric–enzymatic assay (nitrite)	[79]
Electrowetting	Switching (ON)	Lateral flow assay (*S. cerevisiae* rRNA)	[80]
Electrowetting	Switching (ON)	Colorimetric–enzymatic assay (KIO_3_)	[81]
Electro-osmotic pumping	Switching (ON, OFF), Fluid velocity control	-	[82]
Temperature	Switching (ON)	Enzymatic assay (Fe^3+^ and SCN^-^)	[83]
Temperature	Switching (ON)	^#^ NAAT (methicillin-resistant *Staphylococcus* *Aureus*; MRSA bacteria)	[84]
Temperature	Switching (ON, OFF)	Signal enhancement assay (*Escherichia coli*)	[85]

^#^ NAAT: Nucleic acid amplification test.

**Table 4:** Geometry-based active fluid manipulation techniques for μPADs.

Method	Function	Application	Ref.
Cut switch	Switching (ON)	-	[18]
Folding	Switching (ON)	Cell lysis and DNA extraction (*Escherichia* c*oli*)	[4]
Folding	Switching (ON)	Photoelectrochemical detection (Target-ssDNA)	[86]
Folding	Switching (ON)	Electrochemical ELISA (malaria protein PfHRP2)	[87]
Folding	Switching (ON)	Chemiluminescence (CL) detection (*Listeria monocytogenes* hlyA gene)	[37]
Folding	Switching (ON)	Electrochemical enzymatic assay (methyl parathion)	[88]
Folding	Switching (ON)	Electrochemical ELISA (p24 antigen)	[89]
Folding	Switching (ON)	Colorimetric–enzymatic assay (Phe)	[90]
Folding (pop-up)	Switching (ON)	Electrochemical enzymatic assay (Glucose and beta-hydroxybutyrate; BHB)	[91]
Folding (pop-up)	Switching (ON)	Colorimetric–enzymatic assay (acetylcholinesterases; AChE)	[29]
Folding	Switching (ON)	Colorimetric–enzymatic assay (protein A)	[92]
Push-button	Switching (ON)	Colorimetric–enzymatic assay (glucose, ketones, nitrite, and protein)	[93]
Sliding action	Switching (ON)	Colorimetric–enzymatic assay (glucose and protein)	[94]
Sliding action	Switching (ON)	Electrochemical detection (MnO_4_^-^)	[95]
Sliding action	Switching (ON)	NAAT (*Escherichia coli malB* gene)	[96]
Sliding action	Switching (ON)	Electrochemical detection (Ricin)	[97]
Sliding action	Switching (ON)	Electrochemical detection (hepatitis B virus; HBV DNA)	[98]
Sliding action	Switching (ON)	Electrochemical ELISA (Trefoil Factor 3)	[99]
Sliding action	Switching (ON)	Signal enhancement assay (human norovirus)	[100]
Sliding action	Switching (ON)	ELISA (C-reactive protein; CRP)	[36]
Rotational valve (circular disk)	Switching (ON, OFF, diversion)	Signal enhancement assay (*Escherichia coli* O157:H7)	[101]
Rotational valve (hollow rivet)	Switching (ON)	ELISA (carcino-embryonic antigen; CEA)	[102]
Rotational valve (comb binding)	Switching (ON)	Colorimetric–enzymatic assay (Fe^2+^ and nitrite)	[103]
Rotational valve (circular disk)	Switching (ON)	Fluorescence-based molecular-imprinting detection (4-nitrophenol and 2,4,6-trinitrophenol)	[39]

**Table 5:** Mechanical actuation-based active fluid manipulation techniques for μPADs.

Method	Function	Application	Ref.
Pressure valve	Switching (ON, OFF), Fluid velocity control	ELISA (mouse IgG)	[48]
Expandable material	Switching (ON, OFF, diversion)	Signal enhancement assay (malaria protein PfHRP2)	[104]
Magnetic valve	Switching (ON, OFF)	Enzyme-based colorimetric detection (ALP)	[105]
Magnetic valve	Switching (ON, OFF)	-	[106]
Reconfigurable flow switch	Switching (ON, OFF, diversion)	Colorimetric–enzymatic assay (glucose, protein, and nitrite)	[107]

**Table 6:** μPAD technologies for nucleic acid amplification testing.

Method	Analyte	Detection Limit (μPAD)	Detection Limit (Conventional Method)
Temperature	Methicillin-resistant *Staphylococcus aureus*; MRSA bacteria	5 × 10^3^ genomic copies [84]	^#1^ LAMP, 10 genomic copies [114]
Folding	*Escherichia coli*	33 CFU mL^−1^ [4]	^#2^ SERS, 8 CFU mL^−1^ [115]
Sliding action	*Escherichia coli* malB gene	500 cells mL^−1^ [96]	LAMP, 1.02 copies [116]
Sliding action	*Salmonella typhimurium*	10^2^ CFU mL^−1^ [111]	^#3^ QCM, 10^0^ CFU mL^−1^ [117]
Folding	Influenza A (H1N1)	10^6^ copies mL^−1^ [113]	RT-PCR, 384 copies mL^−1^ [118]
Folding	Human papillomavirus (HPV) 16 DNA	10^4^ copies [112]	qPCR, 1.65 copies [119]

^#1^ LAMP: Loop-mediated isothermal amplification; ^#2^ SERS: Surface-enhanced Raman scattering; ^#3^ QCM: Quartz crystal microbalance.

**Table 7:** μPAD technologies for enzyme-linked immunosorbent assay.

Method	Analyte	Detection Limit (μPAD)	Detection Limit (Conventional Method)
Inkjet printing	Human chorionic gonadotropin; hCG	1 ng mL^−1^ [55]	Electrochemical immunoassay, 20 pM in human urine [123]
Inkjet printing	Alpha-fetoprotein; AFP	1 ng mL^−1^ [120]	Sandwich immunoassay, 3.4 ng mL^−1^ [124]
Surfactant	Rabbit IgG	4.8 fM [58]	ELISA, 3.4 ng mL^−1^ [125]
Folding	Malaria protein PfHRP2	4 ng mL^−1^ [87]	ELISA, 2.5 pg mL^−1^ [126]
Folding	Human immunodeficiency virus p24 antigen	300 fg mL^−1^ [89]	Sandwich immunoassay, 10^−17^ g mL^−1^ [127]
Sliding action	Trefoil factor 3	12.5 ng mL^−1^ [99]	ELISA, 3 pM [128]
Sliding action	C-reactive protein; CRP	1 ng mL^−1^ [36]	Immunonephelometric assay, 0.17 mg L^−1^ [129]
Rotational valve (hollow rivet)	Carcino-embryonic antigen; CEA	0.3 ng mL^−1^ [102]	dsDNA-templated ^#^ CuNPs coupled with a CEA-specific aptamer, 0.0065 ng mL^−1^ [130]
Pressure valve	Mouse IgG	50 ng mL^−1^ [48]	Sandwich immunoassay, 15.6 ng mL^−1^ [131]

^#^ CuNP: Copper nanoparticle.

**Table 8:** μPAD technologies for signal enhancement assay (gold enhancement).

Method	Analyte	Amplification Ratio	Ref.
Dissolvable material (Sucrose)	PfHRP2 malaria	2.6-fold	[49]
Shunt	Malaria protein PfHRP2	Not marked	[63]
Pressurized paper	Progesterone receptor	4.3-fold	[67]
Pressurized paper	*Escherichia coli* O157:H7 and *Salmonella typhimurium*	10-fold	[68]
Pressurized paper (with 3D stacking)	C-reactive protein	3.47-fold	[69]
Source pad of different sizes (2DPNs)	BSA-biotin	7.3-fold	[20]
Source pad of different sizes (2DPNs)	Malaria protein PfHRP2	4-fold	[70]
Source pad of different sizes (2DPNs)	Malaria protein PfHRP2	3.2-fold	[71]
Temperature	*Escherichia coli*	6-fold	[85]
Sliding action	Human norovirus	3-fold	[100]
Expandable material	Malaria protein PfHRP2	Not marked	[104]

**Table 9:** μPAD technologies for colorimetric enzymatic assay.

Method	Analyte	Detection Limit (μPAD)	Detection Limit (Conventional Method)
Dissolvable bridge (pullulan)	Malathion	6 nM [32]	Enzyme assay, 0.3 nM [137]
Covered film	Malathion	75 nM [33]	
Target-responsive hydrogel	Cocaine	7.3 μM [60]	Mass spectrometry, 30 ng mL^−1^ [138]
Hollow channel	Glucose/BSA	0.7 mM/18 μM [34]	ELISA, 32 ng mL^−1^ [139]
Sliding action	BSA	4.8 nM [94]	
Corona discharge treatment	Nitrite	7.8 μmol L^−^^1^ [79]	Griess assay, 0.02 µM [140]
Rotational valve (comb binding)	Fe^2+^/nitrite	8.9/0.28 mg L^−^^1^ [103]	

## References

[1] Dungchai W., Chailapakul O., Henry C.S. (2010). Use of multiple colorimetric indicators for paper-based microfluidic devices. Anal. Chim. Acta.

[2] Martinez A.W., Phillips S.T., Butte M.J., Whitesides G.M. (2007). Patterned paper as a platform for inexpensive, low-volume, portable bioassays. Angew. Chem. Int. Ed. Engl.

[3] Wang S., Ge L., Song X., Yu J., Ge S., Huang J., Zeng F. (2012). Paper-based chemiluminescence ELISA: Lab-on-paper based on chitosan modified paper device and wax-screen-printing. Biosens. Bioelectron..

[4] Govindarajan A.V., Ramachandran S., Vigil G.D., Yager P., Bohringer K.F. (2012). A low cost point-of-care viscous sample preparation device for molecular diagnosis in the developing world; an example of microfluidic origami. Lab Chip.

[5] Jiang X., Fan Z.H. (2016). Fabrication and Operation of Paper-Based Analytical Devices. Annu. Rev. Anal. Chem..

[6] He Y., Wu Y., Fu J.Z., Wu W.B. (2015). Fabrication of paper-based microfluidic analysis devices: A review. RSC Adv..

[7] Akyazi T., Basabe-Desmonts L., Benito-Lopez F. (2018). Review on microfluidic paper-based analytical devices towards commercialisation. Anal. Chim. Acta.

[8] Lee V.B.C., Mohd-Naim N.F., Tamiya E., Ahmed M.U. (2018). Trends in Paper-based Electrochemical Biosensors: From Design to Application. Anal. Sci..

[9] Yamada K., Shibata H., Suzuki K., Citterio D. (2017). Toward practical application of paper-based microfluidics for medical diagnostics: State-of-the-art and challenges. Lab Chip.

[10] Kirk K.A., Othman A., Andreescu S. (2018). Nanomaterial-functionalized Cellulose: Design, Characterization and Analytical Applications. Anal. Sci..

[11] Lim H., Jafry A.T., Lee K. (2019). Fabrication, Flow Control, and Applications of Microfluidic Paper-Based Analytical Devices. Molecules.

[12] Fu E., Downs C. (2017). Progress in the development and integration of fluid flow control tools in paper microfluidics. Lab Chip.

[13] Martinez A.W., Phillips S.T., Wiley B.J., Whitesides G.M. (2008). FLASH: A rapid method for prototyping paper-based microfluidic devices. Lab Chip.

[14] Yu W.W., White I.M. (2010). Inkjet printed surface enhanced raman spectroscopy array on cellulose paper. Anal. Chem..

[15] Xu C., Cai L., Zhong M., Zheng S. (2015). Low-cost and rapid prototyping of microfluidic paper-based analytical devices by inkjet printing of permanent marker ink. RSC Adv..

[16] Lu Y., Shi W., Qin J., Lin B. (2010). Fabrication and Characterization of Paper-Based Microfluidics Prepared in nitrocellulose membrane by wax printing. Anal. Chem..

[17] Cai L., Wu Y., Xu C., Chen Z. (2012). A Simple Paper-Based Microfluidic Device for the Determination of the Total Amino Acid Content in a Tea Leaf Extract. J. Chem. Educ..

[18] Li X., Tian J., Nguyen T., Shen W. (2008). Paper-based microfluidic devices by plasma treatment. Anal. Chem..

[19] Fenton E.M., Mascarenas M.R., Lopez G.P., Sibbett S.S. (2009). Multiplex lateral-flow test strips fabricated by two-dimensional shaping. ACS Appl. Mater. Interfaces.

[20] Fu E., Kauffman P., Lutz B., Yager P. (2010). Chemical signal amplification in two-dimensional paper networks. Sens. Actuators B.

[21] Dungchai W., Chailapakul O., Henry C.S. (2011). A low-cost, simple, and rapid fabrication method for paper-based microfluidics using wax screen-printing. Analyst.

[22] Sun J.Y., Cheng C.M., Liao Y.C. (2015). Screen printed paper-based diagnostic devices with polymeric inks. Anal. Sci..

[23] Chitnis G., Ding Z., Chang C.L., Savran C.A., Ziaie B. (2011). Laser-treated hydrophobic paper: An inexpensive microfluidic platform. Lab Chip.

[24] Olkkonen J., Lehtinen K., Erho T. (2010). Flexographically Printed Fluidic Structures in Paper. Anal. Chem..

[25] Määttänen A., Fors D., Wang S., Valtakari D., Ihalainen P., Peltonen J. (2011). Paper-based planar reaction arrays for printed diagnostics. Sens. Actuators B.

[26] Dou M., Sanjay S.T., Benhabib M., Xu F., Li X. (2015). Low-cost bioanalysis on paper-based and its hybrid microfluidic platforms. Talanta.

[27] Shih C.M., Chang C.L., Hsu M.Y., Lin J.Y., Kuan C.M., Wang H.K., Huang C.T., Chung M.C., Huang K.C., Hsu C.E. (2015). Paper-based ELISA to rapidly detect Escherichia coli. Talanta.

[28] Meredith N.A., Quinn C., Cate D.M., Reilly T.H., Volckens J., Henry C.S. (2016). Paper-based analytical devices for environmental analysis. Analyst.

[29] Lee S., Park J., Park J.K. (2018). Foldable paper-based analytical device for the detection of an acetylcholinesterase inhibitor using an angle-based readout. Sens. Actuators B.

[30] Wang H., Wang J., Timchalk C., Lin Y. (2008). Magnetic electrochemical immunoassays with quantum dot labels for detection of phosphorylated acetylcholinesterase in plasma. Anal. Chem..

[31] Ansari N., Lodha A., Pandya A., Menon S.K. (2017). Determination of cause of death using paper-based microfluidic device as a colorimetric probe. Anal. Methods.

[32] Jahanshahi-Anbuhi S., Henry A., Leung V., Sicard C., Pennings K., Pelton R., Brennan J.D., Filipe C.D. (2014). Paper-based microfluidics with an erodible polymeric bridge giving controlled release and timed flow shutoff. Lab Chip.

[33] Jahanshahi-Anbuhi S., Chavan P., Sicard C., Leung V., Hossain S.M., Pelton R., Brennan J.D., Filipe C.D. (2012). Creating fast flow channels in paper fluidic devices to control timing of sequential reactions. Lab Chip.

[34] Renault C., Li X., Fosdick S.E., Crooks R.M. (2013). Hollow-channel paper analytical devices. Anal. Chem..

[35] Giokas D.L., Tsogas G.Z., Vlessidis A.G. (2014). Programming fluid transport in paper-based microfluidic devices using razor-crafted open channels. Anal. Chem..

[36] Verma M.S., Tsaloglou M.N., Sisley T., Christodouleas D., Chen A., Milette J., Whitesides G.M. (2018). Sliding-strip microfluidic device enables ELISA on paper. Biosens. Bioelectron..

[37] Liu F., Zhang C. (2015). A novel paper-based microfluidic enhanced chemiluminescence biosensor for facile, reliable and highly-sensitive gene detection of Listeria monocytogenes. Sens. Actuators B.

[38] Renault C., Anderson M.J., Crooks R.M. (2014). Electrochemistry in hollow-channel paper analytical devices. J. Am. Chem. Soc..

[39] Qi J., Li B., Wang X., Fu L., Luo L., Chen L. (2018). Rotational Paper-Based Microfluidic-Chip Device for Multiplexed and Simultaneous Fluorescence Detection of Phenolic Pollutants Based on a Molecular-Imprinting Technique. Anal. Chem..

[40] Wu L., Ma C., Zheng X., Liu H., Yu J. (2015). Paper-based electrochemiluminescence origami device for protein detection using assembled cascade DNA-carbon dots nanotags based on rolling circle amplification. Biosens. Bioelectron..

[41] Strong E.B., Knutsen C., Wells J.T., Jangid A.R., Mitchell M.L., Martinez N.W., Martinez A.W. (2019). Wax-Printed Fluidic Time Delays for Automating Multi-Step Assays in Paper-Based Microfluidic Devices (MicroPADs). Inventions.

[42] Washburn E.W. (1921). The Dynamics of Capillary Flow. Phys. Rev..

[43] Lucas R. (2018). Ueber das Zeitgesetz des kapillaren Aufstiegs von Flüssigkeiten. Kolloid Z..

[44] Mendez S., Fenton E.M., Gallegos G.R., Petsev D.N., Sibbett S.S., Stone H.A., Zhang Y., Lopez G.P. (2010). Imbibition in porous membranes of complex shape: Quasi-stationary flow in thin rectangular segments. Langmuir.

[45] Soum V., Park S., Brilian A.I., Kwon O.S., Shin K. (2019). Programmable Paper-Based Microfluidic Devices for Biomarker Detections. Micromachines.

[46] Fu E., Ramsey S.A., Kauffman P., Lutz B., Yager P. (2011). Transport in two-dimensional paper networks. Microfluid. Nanofluid..

[47] Chen H., Cogswell J., Anagnostopoulos C., Faghri M. (2012). A fluidic diode, valves, and a sequential-loading circuit fabricated on layered paper. Lab Chip.

[48] Kim T.H., Hahn Y.K., Lee J., van Noort D., Kim M.S. (2018). Solenoid Driven Pressure Valve System: Toward Versatile Fluidic Control in Paper Microfluidics. Anal. Chem..

[49] Lutz B., Liang T., Fu E., Ramachandran S., Kauffman P., Yager P. (2013). Dissolvable fluidic time delays for programming multi-step assays in instrument-free paper diagnostics. Lab Chip.

[50] Fu E., Lutz B., Kauffman P., Yager P. (2010). Controlled reagent transport in disposable 2D paper networks. Lab Chip.

[51] Schilling K.M., Lepore A.L., Kurian J.A., Martinez A.W. (2012). Fully enclosed microfluidic paper-based analytical devices. Anal. Chem..

[52] Noh H., Phillips S.T. (2010). Metering the Capillary-Driven Flow of Fluids in paper-based microfluidic devices. Anal. Chem..

[53] Noh H., Phillips S.T. (2010). Fluidic Timers for Time-Dependent, Point-of-Care Assays on Paper. Anal. Chem..

[54] Weng C.H., Chen M.Y., Shen C.H., Yang R.J. (2014). Colored wax-printed timers for two-dimensional and three-dimensional assays on paper-based devices. Biomicrofluidics.

[55] Apilux A., Ukita Y., Chikae M., Chailapakul O., Takamura Y. (2013). Development of automated paper-based devices for sequential multistep sandwich enzyme-linked immunosorbent assays using inkjet printing. Lab Chip.

[56] Houghtaling J., Liang T., Thiessen G., Fu E. (2013). Dissolvable bridges for manipulating fluid volumes in paper networks. Anal. Chem..

[57] Salentijn G.I., Hamidon N.N., Verpoorte E. (2016). Solvent-dependent on/off valving using selectively permeable barriers in paper microfluidics. Lab Chip.

[58] Gerbers R., Foellscher W., Chen H., Anagnostopoulos C., Faghri M. (2014). A new paper-based platform technology for point-of-care diagnostics. Lab Chip.

[59] Wei X., Tian T., Jia S., Zhu Z., Ma Y., Sun J., Lin Z., Yang C.J. (2015). Target-responsive DNA hydrogel mediated "stop-flow" microfluidic paper-based analytic device for rapid, portable and visual detection of multiple targets. Anal. Chem..

[60] Tian T., Wei X., Jia S., Zhang R., Li J., Zhu Z., Zhang H., Ma Y., Lin Z., Yang C.J. (2016). Integration of target responsive hydrogel with cascaded enzymatic reactions and microfluidic paper-based analytic devices (microPADs) for point-of-care testing (POCT). Biosens. Bioelectron..

[61] Chu W., Chen Y., Liu W., Zhao M., Li H. (2017). Paper-based chemiluminescence immunodevice with temporal controls of reagent transport technique. Sens. Actuators B.

[62] Songok J., Toivakka M. (2016). Controlling capillary-driven surface flow on a paper-based microfluidic channel. Microfluid. Nanofluid..

[63] Toley B.J., McKenzie B., Liang T., Buser J.R., Yager P., Fu E. (2013). Tunable-delay shunts for paper microfluidic devices. Anal. Chem..

[64] Glavan A.C., Martinez R.V., Maxwell E.J., Subramaniam A.B., Nunes R.M., Soh S., Whitesides G.M. (2013). Rapid fabrication of pressure-driven open-channel microfluidic devices in omniphobic R^F^ paper. Lab Chip.

[65] da Silva E.T., Santhiago M., de Souza F.R., Coltro W.K., Kubota L.T. (2015). Triboelectric effect as a new strategy for sealing and controlling the flow in paper-based devices. Lab Chip.

[66] Shin J.H., Lee G.J., Kim W., Choi S. (2016). A stand-alone pressure-driven 3D microfluidic chemical sensing analytic device. Sensor Actuat B-Chem.

[67] Shin J.H., Park J., Kim S.H., Park J.K. (2014). Programmed sample delivery on a pressurized paper. Biomicrofluidics.

[68] Park J., Shin J.H., Park J.K. (2016). Pressed Paper-Based Dipstick for Detection of Foodborne Pathogens with Multistep Reactions. Anal. Chem..

[69] Park J., Park J.K. (2017). Pressed region integrated 3D paper-based microfluidic device that enables vertical flow multistep assays for the detection of C-reactive protein based on programmed reagent loading. Sens. Actuators B.

[70] Fu E., Liang T., Spicar-Mihalic P., Houghtaling J., Ramachandran S., Yager P. (2012). Two-dimensional paper network format that enables simple multistep assays for use in low-resource settings in the context of malaria antigen detection. Anal. Chem..

[71] Fridley G.E., Le H., Yager P. (2014). Highly sensitive immunoassay based on controlled rehydration of patterned reagents in a 2-dimensional paper network. Anal. Chem..

[72] Martinez A.W., Phillips S.T., Whitesides G.M. (2008). Three-dimensional microfluidic devices fabricated in layered paper and tape. Proc. Natl. Acad. Sci. USA.

[73] Liu H., Crooks R.M. (2011). Three-dimensional paper microfluidic devices assembled using the principles of origami. J. Am. Chem. Soc..

[74] Deraney R.N., Mace C.R., Rolland J.P., Schonhorn J.E. (2016). Multiplexed, Patterned-Paper Immunoassay for Detection of Malaria and Dengue Fever. Anal. Chem..

[75] Morbioli G.G., Mazzu-Nascimento T., Milan L.A., Stockton A.M., Carrilho E. (2017). Improving Sample Distribution Homogeneity in Three-Dimensional Microfluidic Paper-Based Analytical Devices by Rational Device Design. Anal. Chem..

[76] Lutz B.R., Trinh P., Ball C., Fu E., Yager P. (2011). Two-dimensional paper networks: Programmable fluidic disconnects for multi-step processes in shaped paper. Lab Chip.

[77] Jeong S.G., Lee S.H., Choi C.H., Kim J., Lee C.S. (2015). Toward instrument-free digital measurements: A three-dimensional microfluidic device fabricated in a single sheet of paper by double-sided printing and lamination. Lab Chip.

[78] Matsuda Y., Shibayama S., Uete K., Yamaguchi H., Niimi T. (2015). Electric conductive pattern element fabricated using commercial inkjet printer for paper-based analytical devices. Anal Chem.

[79] Jiang Y., Hao Z., He Q., Chen H. (2016). A simple method for fabrication of microfluidic paper-based analytical devices and on-device fluid control with a portable corona generator. RSC Adv..

[80] Koo C.K., He F., Nugen S.R. (2013). An inkjet-printed electrowetting valve for paper-fluidic sensors. Analyst.

[81] Ainla A., Hamedi M.M., Guder F., Whitesides G.M. (2017). Electrical Textile Valves for Paper Microfluidics. Adv. Mater..

[82] Rosenfeld T., Bercovici M. (2019). Dynamic control of capillary flow in porous media by electroosmotic pumping. Lab Chip.

[83] Cai L., Zhong M., Li H., Xu C., Yuan B. (2015). Defining microchannels and valves on a hydrophobic paper by low-cost inkjet printing of aqueous or weak organic solutions. Biomicrofluidics.

[84] Lafleur L.K., Bishop J.D., Heiniger E.K., Gallagher R.P., Wheeler M.D., Kauffman P., Zhang X., Kline E.C., Buser J.R., Kumar S. (2016). A rapid, instrument-free, sample-to-result nucleic acid amplification test. Lab Chip.

[85] Phillips E.A., Shen R., Zhao S., Linnes J.C. (2016). Thermally actuated wax valves for paper-fluidic diagnostics. Lab Chip.

[86] Wang Y., Ge L., Wang P., Yan M., Ge S., Li N., Yu J., Huang J. (2013). Photoelectrochemical lab-on-paper device equipped with a porous Au-paper electrode and fluidic delay-switch for sensitive detection of DNA hybridization. Lab Chip.

[87] Glavan A.C., Christodouleas D.C., Mosadegh B., Yu H.D., Smith B.S., Lessing J., Fernandez-Abedul M.T., Whitesides G.M. (2014). Folding analytical devices for electrochemical ELISA in hydrophobic R^H^ paper. Anal. Chem..

[88] Ding J., Li B., Chen L., Qin W. (2016). A Three-Dimensional Origami Paper-Based Device for Potentiometric Biosensing. Angew. Chem. Int. Ed. Engl..

[89] Li X., Liu X. (2016). A Microfluidic Paper-Based Origami Nanobiosensor for Label-Free, Ultrasensitive Immunoassays. Adv. Healthc. Mater..

[90] Robinson R., Wong L., Monnat R.J., Fu E. (2016). Development of a Whole Blood Paper-Based Device for Phenylalanine Detection in the Context of PKU Therapy Monitoring. Micromachines.

[91] Wang C.C., Hennek J.W., Ainla A., Kumar A.A., Lan W.J., Im J., Smith B.S., Zhao M., Whitesides G.M. (2016). A Paper-Based “Pop-up” Electrochemical Device for Analysis of Beta-Hydroxybutyrate. Anal. Chem..

[92] Chen C.A., Yeh W.S., Tsai T.T., Li Y.D., Chen C.F. (2019). Three-dimensional origami paper-based device for portable immunoassay applications. Lab Chip.

[93] Martinez A.W., Phillips S.T., Nie Z., Cheng C.M., Carrilho E., Wiley B.J., Whitesides G.M. (2010). Programmable diagnostic devices made from paper and tape. Lab Chip.

[94] Liu H., Li X., Crooks R.M. (2013). Paper-based SlipPAD for high-throughput chemical sensing. Anal. Chem..

[95] Scida K., Cunningham J.C., Renault C., Richards I., Crooks R.M. (2014). Simple, sensitive, and quantitative electrochemical detection method for paper analytical devices. Anal. Chem..

[96] Connelly J.T., Rolland J.P., Whitesides G.M. (2015). “Paper Machine” for Molecular Diagnostics. Anal. Chem..

[97] Cunningham J.C., Scida K., Kogan M.R., Wang B., Ellington A.D., Crooks R.M. (2015). Paper diagnostic device for quantitative electrochemical detection of ricin at picomolar levels. Lab Chip.

[98] Li X., Scida K., Crooks R.M. (2015). Detection of hepatitis B virus DNA with a paper electrochemical sensor. Anal. Chem..

[99] DeGregory P.R., Tsai Y.J., Scida K., Richards I., Crooks R.M. (2016). Quantitative electrochemical metalloimmunoassay for TFF3 in urine using a paper analytical device. Analyst.

[100] Han K.N., Choi J.S., Kwon J. (2016). Three-dimensional paper-based slip device for one-step point-of-care testing. Sci. Rep..

[101] Shin J.H., Park J.K. (2016). Functional Packaging of Lateral Flow Strip Allows Simple Delivery of Multiple Reagents for Multistep Assays. Anal. Chem..

[102] Li B., Yu L., Qi J., Fu L., Zhang P., Chen L. (2017). Controlling Capillary-Driven Fluid Transport in Paper-Based Microfluidic Devices Using a Movable Valve. Anal. Chem..

[103] Han J., Qi A., Zhou J., Wang G., Li B., Chen L. (2018). Simple Way To Fabricate Novel Paper-Based Valves Using Plastic Comb Binding Spines. ACS Sens..

[104] Toley B.J., Wang J.A., Gupta M., Buser J.R., Lafleur L.K., Lutz B.R., Fu E., Yager P. (2015). A versatile valving toolkit for automating fluidic operations in paper microfluidic devices. Lab Chip.

[105] Li X., Zwanenburg P., Liu X. (2013). Magnetic timing valves for fluid control in paper-based microfluidics. Lab Chip.

[106] Fratzl M., Chang B.S., Oyola-Reynoso S., Blaire G., Delshadi S., Devillers T., Ward T., Dempsey N.M., Bloch J.F., Thuo M.M. (2018). Magnetic Two-Way Valves for Paper-Based Capillary-Driven Microfluidic Devices. ACS Omega.

[107] Kong T., Flanigan S., Weinstein M., Kalwa U., Legner C., Pandey S. (2017). A fast, reconfigurable flow switch for paper microfluidics based on selective wetting of folded paper actuator strips. Lab Chip.

[108] Choi J.R., Hu J., Gong Y., Feng S., Wan Abas W.A., Pingguan-Murphy B., Xu F. (2016). An integrated lateral flow assay for effective DNA amplification and detection at the point of care. Analyst.

[109] Linnes J.C., Fan A., Rodriguez N.M., Lemieux B., Kong H., Klapperich C.M. (2014). Paper-based molecular diagnostic for Chlamydia trachomatis. RSC Adv..

[110] Cordray M.S., Richards-Kortum R.R. (2015). A paper and plastic device for the combined isothermal amplification and lateral flow detection of Plasmodium DNA. Malar. J..

[111] Tang R., Yang H., Gong Y., You M., Liu Z., Choi J.R., Wen T., Qu Z., Mei Q., Xu F. (2017). A fully disposable and integrated paper-based device for nucleic acid extraction, amplification and detection. Lab Chip.

[112] Rodriguez N.M., Wong W.S., Liu L., Dewar R., Klapperich C.M. (2016). A fully integrated paperfluidic molecular diagnostic chip for the extraction, amplification, and detection of nucleic acids from clinical samples. Lab Chip.

[113] Rodriguez N.M., Linnes J.C., Fan A., Ellenson C.K., Pollock N.R., Klapperich C.M. (2015). Paper-Based RNA Extraction, in Situ Isothermal Amplification, and Lateral Flow Detection for Low-Cost, Rapid Diagnosis of Influenza A (H1N1) from Clinical Specimens. Anal. Chem..

[114] Su J., Liu X., Cui H., Li Y., Chen D., Li Y., Yu G. (2014). Rapid and simple detection of methicillin-resistance Staphylococcus aureus by orfX loop-mediated isothermal amplification assay. BMC Biotechnol..

[115] Guven B., Basaran-Akgul N., Temur E., Tamer U., Boyaci I.H. (2011). SERS-based sandwich immunoassay using antibody coated magnetic nanoparticles for Escherichia coli enumeration. Analyst.

[116] Ramezani R., Kardoost Parizi Z., Ghorbanmehr N., Mirshafiee H. (2018). Rapid and Simple Detection of Escherichia coli by Loop-Mediated Isothermal Amplification Assay in Urine Specimens. Avicenna. J. Med. Biotechnol..

[117] Fulgione A., Cimafonte M., Della Ventura B., Iannaccone M., Ambrosino C., Capuano F., Proroga Y.T.R., Velotta R., Capparelli R. (2018). QCM-based immunosensor for rapid detection of Salmonella Typhimurium in food. Sci. Rep..

[118] Panning M., Eickmann M., Landt O., Monazahian M., Olschlager S., Baumgarte S., Reischl U., Wenzel J.J., Niller H.H., Gunther S. (2009). Detection of influenza A(H1N1)v virus by real-time RT-PCR. Eurosurveill..

[119] Shigeishi H., Sugiyama M., Ohta K., Yokoyama S., Sakuma M., Murozumi H., Kato H., Takechi M. (2018). High HPV16 E6 viral load in the oral cavity is associated with an increased number of bacteria: A preliminary study. Biomed. Rep..

[120] Preechakasedkit P., Siangproh W., Khongchareonporn N., Ngamrojanavanich N., Chailapakul O. (2018). Development of an automated wax-printed paper-based lateral flow device for alpha-fetoprotein enzyme-linked immunosorbent assay. Biosens. Bioelectron..

[121] Grant B.D., Smith C.A., Karvonen K., Richards-Kortum R. (2016). Highly Sensitive Two-Dimensional Paper Network Incorporating Biotin-Streptavidin for the Detection of Malaria. Anal. Chem..

[122] Ramachandran S., Fu E., Lutz B., Yager P. (2014). Long-term dry storage of an enzyme-based reagent system for ELISA in point-of-care devices. Analyst.

[123] Kerman K., Nagatani N., Chikae M., Yuhi T., Takamura Y., Tamiya E. (2006). Label-free electrochemical immunoassay for the detection of human chorionic gonadotropin hormone. Anal. Chem..

[124] Wang Q., Li R., Shao K., Lin Y., Yang W., Guo L., Qiu B., Lin Z., Chen G. (2017). A Portable Immunosensor with Differential Pressure Gauges Readout for Alpha Fetoprotein Detection. Sci. Rep..

[125] Tsai H., Lu Y.H., Liao H.X., Wu S.W., Yu F.Y., Fuh C.B. (2015). Detection of rabbit IgG by using functional magnetic particles and an enzyme-conjugated antibody with a homemade magnetic microplate. Chem. Cent. J..

[126] Soraya G.V., Abeyrathne C.D., Buffet C., Huynh D.H., Uddin S.M., Chan J., Skafidas E., Kwan P., Rogerson S.J. (2019). Ultrasensitive and label-free biosensor for the detection of Plasmodium falciparum histidine-rich protein II in saliva. Sci. Rep..

[127] Kosaka P.M., Pini V., Calleja M., Tamayo J. (2017). Ultrasensitive detection of HIV-1 p24 antigen by a hybrid nanomechanical-optoplasmonic platform with potential for detecting HIV-1 at first week after infection. PLoS ONE.

[128] Vestergaard E.M., Poulsen S.S., Gronbaek H., Larsen R., Nielsen A.M., Ejskjaer K., Clausen J.T., Thim L., Nexo E. (2002). Development and evaluation of an ELISA for human trefoil factor 3. Clin. Chem..

[129] Khuseyinova N., Imhof A., Trischler G., Rothenbacher D., Hutchinson W.L., Pepys M.B., Koenig W. (2003). Determination of C-reactive protein: Comparison of three high-sensitivity immunoassays. Clin. Chem..

[130] Chen M., Khusbu F.Y., Ma C., Wu K., Zhao H., Chena H., Wang K. (2018). A sensitive detection method of carcinoembryonic antigen based on dsDNA-templated copper nanoparticles. New J. Chem..

[131] Kim K.S., Park J.K. (2005). Magnetic force-based multiplexed immunoassay using superparamagnetic nanoparticles in microfluidic channel. Lab Chip.

[132] Jung B., Bharadwaj R., Santiago J.G. (2006). On-chip millionfold sample stacking using transient isotachophoresis. Anal. Chem..

[133] Li X., Luo L., Crooks R.M. (2015). Low-voltage paper isotachophoresis device for DNA focusing. Lab Chip.

[134] Xu C., Zhong M., Cai L., Zheng Q., Zhang X. (2016). Sample injection and electrophoretic separation on a simple laminated paper based analytical device. Electrophoresis.

[135] Ge L., Wang S., Ge S., Yu J., Yan M., Li N., Huang J. (2014). Electrophoretic separation in a microfluidic paper-based analytical device with an on-column wireless electrogenerated chemiluminescence detector. Chem. Commun..

[136] OuYang L., Wang C., Du F., Zheng T., Liang H. (2014). Electrochromatographic separations of multicomponent metal complexes on a microfluidic paper-based device with a simplified photolithography. RSC Adv..

[137] Rodrigues N.F.M., Neto S.Y., Luz R.C.S., Damos F.S., Yamanaka H. (2018). Ultrasensitive Determination of Malathion Using Acetylcholinesterase Immobilized on Chitosan-Functionalized Magnetic Iron Nanoparticles. Biosensors.

[138] Ismail M., Baumert M., Stevenson D., Watts J., Webb R., Costa C., Robinsonc F., Baileya M. (2017). A diagnostic test for cocaine and benzoylecgonine in urine and oral fluid using portable mass spectrometry. Anal. Methods.

[139] Khamehchian S., Madani R., Golchinfar F., Taghavian M. (2008). Development of a sandwich enzyme-linked immunosorbent assay (ELISA) for determining of bovine serum albumin (BSA) in trivalent measles-mump-rubella (MMR) vaccines. Hum. Vaccin. Immunother..

[140] Beaton A.D., Cardwell C.L., Thomas R.S., Sieben V.J., Legiret F.E., Waugh E.M., Statham P.J., Mowlem M.C., Morgan H. (2012). Lab-on-chip measurement of nitrate and nitrite for in situ analysis of natural waters. Environ. Sci. Technol..

